# Recapitulating the tumor microenvironment in a dish, one cell type at a time

**DOI:** 10.1016/j.crmeth.2024.100800

**Published:** 2024-06-17

**Authors:** Benjamin N. Ostendorf

**Affiliations:** 1 Department of Hematology, Oncology, and Tumor Immunology, Charité-Universitätsmedizin Berlin, Berlin, Germany; 2 Berlin Institute of Health, Berlin, Germany; 3 Berlin Institute for Medical Systems Biology (BIMSB), Max Delbrück Center for Molecular Medicine, Berlin, Germany; 4 German Cancer Consortium (DKTK), Partner Site Berlin, and German Cancer Research Center (DKFZ), Heidelberg, Germany

## Abstract

The tumor microenvironment harbors a variety of different cell types that differentially impact tumor biology. In this issue of *Cell Reports Methods*, Raffo-Romero et al. standardized and optimized 3D tumor organoids to model the interactions between tumor-associated macrophages and tumor cells *in vitro*.

## Main text

Tumors are complex ecosystems that contain many different cell types exhibiting diverse functional states. Importantly, non-tumoral cells in the tumor microenvironment (TME) impact many facets of tumor biology, including tumor angiogenesis, tumor cell migration and metastasis, and anti-tumor immunity.^1^ The complexity of the TME *in vivo* limits the conclusions that can be drawn from *in vitro* models of cancer progression and therapy response. In addition, oftentimes cancer cells cannot be propagated as 2D cultures *in vitro*, thereby limiting the application of these models to model an individual patient’s cancer *ex vivo*.

To overcome several of these limitations, 3D organoid models have been developed in recent years that substantially expand the use of *in vitro* models, including the *in vitro* propagation of previously hard-to-propagate cancers, enabling a more physiological modeling of tumor-matrix interactions and a more faithful recapitulation of the spatial organization of tumors.^2^ Despite these advances, organoid models still do not recapitulate several facets of a tumor *in vivo*. Most importantly, conventional tumor organoids mainly contain tumor cells while lacking the diverse cell types that make up the TME. This represents an important limitation for the prediction of individual therapy responses, since non-tumoral cells in the TME exert a major impact on the efficacy of anti-tumor therapies. As one important example, tumor-associated macrophages (TAMs), which are among the most abundant non-tumoral cells in the TME of many tumor types, play a major role in modulating responses to anti-tumor immunotherapy and to conventional chemotherapy.^3^^,^^4^

Several recent studies have addressed this limitation either by devising autologous organoids that integrate non-tumoral stromal cells from the original TME or by reconstituting organoids with either autologous or allogeneic exogenous non-tumoral cells from other sites, such as the peripheral blood.^5^^,^^6^ These approaches exhibit different advantages by either preserving the composition of the original TME or by providing easier manipulation and expansion of non-tumoral cells prior to co-culture. Studies employing these methods have enabled the *in vitro* modeling of therapy responses including immunotherapy^7^ and thus promise to pave the way toward more faithful prediction of cancer therapy responses. However, one major limitation of these more complex tumor organoids is their lack of standardization. In addition, different cell types exhibit divergent optimal growth conditions *in vitro*, thereby rendering it necessary to optimize culture conditions for individual combinations of tumoral and stromal cells.

In this issue of *Cell Reports Methods*, Raffo-Romero et al.^8^ address these limitations by devising three standardized and optimized approaches for reconstituting patient-derived breast cancer organoids with macrophages. In a first step, the authors optimized a protocol for deriving macrophages from peripheral blood monocytes by systematically assessing different culture and cell detachment approaches. The use of cryopreserved peripheral blood monocytes in the optimized protocol allows for decoupling and later synchronizing the generation of tumor organoids and macrophages derived from the same patient.

Next, the authors established three different approaches for co-culturing these macrophages with breast cancer organoids: one semi-liquid system and two solid-state systems. In preparation for all co-cultures, tumor cells were recovered from Matrigel drops and mixed with dye-labeled macrophages at a fixed ratio. In the first approach, Raffo-Romero et al.^8^ established a semi-liquid model by co-culturing macrophages and tumor cells in medium with 2% Matrigel, thus facilitating the direct interaction between the two cell types. In the two solid co-culture systems, tumor cells were embedded in a Matrigel matrix with macrophages either embedded together with tumor cells within the matrix (termed “inner co-culture”) or layered outside on top of the matrix in culture medium (termed “external co-culture”). The authors then tracked the localization of the macrophages over the course of several days and found that macrophages aggregated around the cancer organoids. Notably, macrophages in the external co-culture infiltrated the matrix and also interacted with tumor organoids, indicating that this approach may be suitable to model the infiltration of the TME by TAMs. Not surprisingly, the proportion of macrophages in the organoid was lower in the external co-culture than in the other two setups, and, importantly, the different degree of infiltration between the setups proved reproducible across experiments. The authors next sought to characterize the three co-culture systems using immunofluorescence light-sheet microscopy. To overcome substantial background noise in the solid-state conditions, the authors integrated a step to clear the organoids using formamide and polyethylene glycol, enabling the accurate identification of organoid-infiltrating macrophages.

It is well established that macrophages adopt specific phenotypes after infiltrating the TME.^9^ To assess to which degree the macrophages derived from peripheral blood monocytes recapitulated these phenotypes upon co-culture with tumor organoids *in vitro*, the authors next performed proteomic profiling comparing macrophages cultured alone with macrophages co-cultured with tumor organoids. These experiments revealed differential upregulation in co-cultured macrophages of many genes implicated in pathways known to be highly expressed in TAMs. Thus, these results indicate that co-culturing macrophages derived from peripheral blood monocytes in the systems devised by the authors elicits several of the same gene expression programs that are known to be upregulated in TAMs *in vivo*. Interestingly, phenotypic analysis of the expression of prototypical anti-inflammatory “M2-like” markers on macrophages by flow cytometry revealed subtle differences between the three systems, indicating that the specific co-culture implementation imparts differential effects on canonical TAM phenotypes.

The authors next sought to characterize the impact of the presence of TAMs on the molecular profile of the co-cultured tumor organoids. To this end, they optimized a protocol to perform spatially resolved lipidomics on organoids grown in the two solid-state systems to show that the presence of macrophages modulated the lipid profiles of tumor organoids, including enhanced signals for ceramide and lysophospholipid-related lipids.

One of the clinically most salient applications of patient-derived organoids is the prediction of therapy responses in the framework of precision medicine.^10^ Having established and optimized the breast cancer-macrophage co-culture systems, the authors finally sought to characterize the impact of TAMs on chemotherapy response of breast cancer organoids. To this end, they harnessed the semi-liquid system and determined the susceptibility of tumor organoids to paclitaxel, a chemotherapeutic drug commonly used in the treatment of breast cancer. Notably, organoids cultured in the absence of TAMs were more susceptible to paclitaxel-mediated toxicity. These data indicate that the 3D co-culture recapitulates the known effect of TAMs in shielding breast cancer cells from paclitaxel-induced cell death.^11^

The exploration of three different systems for co-culturing macrophages with breast cancer organoids by Raffo-Romero et al.^8^ highlights the different strengths of specific co-culture approaches depending on the question at hand. While the semi-liquid system proved suited best for 3D imaging the entire co-culture as well as for easily recovering cells for phenotypic assessments, the solid-state approaches were better suited for the interrogation of cell-matrix interactions and for modeling TAM infiltration into the TME. More generally, the study by Raffo-Romero et al.^8^ illustrates the benefit of careful optimization and standardization of many steps of more complex organoid systems comprising different cell types.

It is intriguing to envision future applications and extensions of the protocols presented in the study by Raffo-Romero et al.^8^ For example, it is well established that TAMs are an important target of checkpoint immunotherapy.^12^ Therefore, it would be insightful to establish the additional integration of T cells into the dual co-culture systems by applying similarly standardized approaches. It would also be interesting to directly compare the impact of monocyte-derived macrophages on therapy response as performed here to TAMs derived from the autologous TME. Of note, the possibility for decoupled generation of macrophages and tumor organoids employed by the authors would also allow for experimental perturbation of both the tumor or immune cell compartments.

Overall, the study by Raffo-Romero et al.^8^ marks an important advance to model the impact of TAMs on treatment responses of breast cancer cells *in vitro* by providing an optimized and standardized system suitable for multiple phenotypic readouts. More generally, the study lays out a systematic approach for designing and optimizing complex organoid systems comprising multiple cell types.
